# Lessons from integrated seasonal forecast-crop modelling in Africa: A systematic review

**DOI:** 10.1515/biol-2022-0507

**Published:** 2022-11-07

**Authors:** Siyabusa Mkuhlani, Nkulumo Zinyengere, Naomi Kumi, Olivier Crespo

**Affiliations:** Climate Systems Analysis Group, Department of Environmental and Geographical Science, University of Cape Town, Rondebosch, Cape Town 7700, South Africa; Central African Hub, International Institute for Tropical Agriculture (IITA), c/o ICIPE, P. O. Box 30772-00100, Nairobi, Kenya; Agriculture and Food Global Practice, The World Bank Group, 1818H Str NW, Washington DC, 20433, USA; Department of Atmospheric and Climate Science, University of Energy and Natural Resources (UENR), Sunyani, Ghana

**Keywords:** seasonal forecast, crop model, small scale farmer, climate risk management, farm management practice

## Abstract

Seasonal forecasts coupled with crop models can potentially enhance decision-making in smallholder farming in Africa. The study sought to inform future research through identifying and critiquing crop and climate models, and techniques for integrating seasonal forecast information and crop models. Peer-reviewed articles related to crop modelling and seasonal forecasting were sourced from Google Scholar, Web of Science, AGRIS, and JSTOR. Nineteen articles were selected from a search outcome of 530. About 74% of the studies used mechanistic models, which are favored for climate risk management research as they account for crop management practices. European Centre for Medium-Range Weather Forecasts and European Centre for Medium-Range Weather Forecasts, Hamburg, are the predominant global climate models (GCMs) used across Africa. A range of approaches have been assessed to improve the effectiveness of the connection between seasonal forecast information and mechanistic crop models, which include GCMs, analogue, stochastic disaggregation, and statistical prediction through converting seasonal weather summaries into the daily weather. GCM outputs are produced in a format compatible with mechanistic crop models. Such outputs are critical for researchers to have information on the merits and demerits of tools and approaches on integrating seasonal forecast and crop models. There is however need to widen such research to other regions in Africa, crop, farming systems, and policy.

## 1 Introduction

Smallholder farming produces at least 75–90% of food in Africa [1]. It is however characterised by low input and capital investment, limited farming knowledge and transport costs, poor market access, and low crop and livestock productivity. As a result, at least 20% of smallholder farmers in the region experience low crop productivity, leading to perennial physical and dietary food insecurity [2]. Most smallholder farmers practice rain-fed farming and have highlighted seasonal climate and weather variability as the greatest threat to their livelihood [3].

Sub-Saharan Africa experiences high seasonal rainfall variability [4,5]. The rainfall coefficient of variation of ranges from 20 to 45% across sub-humid to semi-arid agro-ecologies [6,7]. As a consequence, rain-fed crop yields range from 15 to 60% relative to mean yield [8]. Crop yield variability has an impact on food security, with impacts being severe amongst resource-constrained and rain-fed-dependent smallholder farming households [3,9].

Seasonal forecast information has the potential to improve farmers’ preparedness to seasonal weather variability. This could enhance seasonal and operational decision-making amongst farmers [10,11,12]. Seasonal forecasts provide information on the magnitude and direction of weather parameters at specific temporal and spatial scales [13], with rainfall and temperature being the key parameters. These parameters are the most significant for agricultural productivity with smallholder farmers being more vulnerable. Using seasonal forecasts, farmers can make seasonal and operational farm management decisions affecting crop and cultivar selection, soil water conservation, planting time, fertiliser application, and harvesting. Despite this potential, the uptake of seasonal forecast information is lower amongst smallholder farmers compared to commercial farmers [14]. One of the key reasons being that farmers do not commonly receive timely and location-specific information, which is relevant to their farming conditions [15]. Seasonal forecasts have relatively low accuracy at longer lead time except in *El Niño* and *La Niña* seasons. On the contrary, forecasts at shorter lead time have relatively high accuracy. Communication of seasonal forecasts in probabilistic terms especially for forecasts at longer lead times also decreases the uptake amongst users [16]. Facilitating and demonstrating the usefulness of coupling seasonal forecasts with crop impact models can support the creation of location-specific, timely and relevant information, thereby increasing the value and chances of uptake by farmers [17,18].

Crop models provide means of conducting quantitative ex ante evaluation of cropping systems’ outputs to given seasonal information [19]. They provide alternate off-field, cost-effective, less complex and less risky means of assessing crop yields and farm management in response to seasonal forecast information [20]. Crop models have been widely used in yield prediction, coupled with seasonal forecast information in the United States of America [21], Europe [22], Australia [23], and east Africa [24]. There is, however, limited research on the use of crop models with seasonal forecasts in Africa [25]. Part of the reason is that coupling seasonal forecast information and crop models can present a variety of complexities. For instance, seasonal forecasts are issued as spatial and temporal summaries over a season and are reported in probabilistic terms [26], whilst most mechanistic crop models require weather data on a daily step [20,27]. This reduces the compatibility between seasonal forecast and crop models for use in crop and climate variability research.

It is important to understand the nature and scope of studies undertaken in Africa, which apply coupled climate forecast information and crop modelling, to better understand the potential for the usefulness of such integrated approaches albeit the inherent challenges and opportunities in enhancing decision-making related to climate variability management. This study reviews previous studies, which integrate seasonal forecast information with crop models for all aspects related to climate risk management. Through the exploration of these published works, the study aims to (1) identify and critique the crop and climate models and the techniques used to integrate seasonal forecast information into crop models in studies related to crop management practices. This will inform future research on the most commonly used tools and approaches and advantages of using such. Future studies on aspects related to seasonal forecasting and crop models will be able to easily identify the most appropriate tools and approaches suitable for African conditions. (2) The study also aims to evaluate changes in crop productivity attributed to the use of seasonal forecast information and crop models in African farming systems.

## 2 Methodology

### 2.1 Systematic literature review

The study used the systematic review approach to explore the scope and state of integrated seasonal forecasting and crop modelling for enhanced decision-making in African smallholder farming systems. The systematic review approach was used as it methodically and critically evaluates literature with the aim of answering certain specific research questions. Traditional literature review is highly vulnerable to personal bias due to pre-conceived knowledge. In contrast, systematic review undertakes a critical review of the studies based on empirical evidence. It is also hinged on planning; undertaking, and reporting of the review outputs and follows a clearly defined comprehensive and repeatable protocol [28].

#### 2.1.1 Research themes

The review was conducted along the following questions:

1. What is the geographical distribution of peer-reviewed research on integration of seasonal forecast information and crop models within Africa?
2. What are the most common sources of seasonal forecast information in Africa?
3. What are the most used crop models in Africa for integration with seasonal forecasts?
4. What techniques are more appropriate for integrating seasonal information and crop models in Africa?
5. What is the intended application of studies integrating seasonal forecast and crop models?
6. What are the subject crops in research involving integrating seasonal forecasts and crop models?
7. What are the range of farm management decisions that are covered by studies integrating seasonal forecast information and crop models?
8. What are the changes in productivity attributed to use of seasonal forecast information?

### 2.2 The systematic review process

The study used peer-reviewed articles, books, and book chapters covering a 15-year period, dating 1 January 2007 to 30 May 2022. The study did not consider grey literature as they were deemed less robust in comparison, due to combined crop and seasonal forecast technical complexity of integration. The original set of peer-reviewed articles was sourced from Google Scholar, Web of Science, JSTOR, and AGRIS online databases and portals, which were found to be exhaustive. These were selected as they have a large reputable database of peer-reviewed literature. The study used specific key words to select articles from the various databases and portals, as shown below.

Google Scholar: “seasonal forecast” AND “crop models” in “Africa.”

Web of Science: seasonal forecast and crop models in Africa.

JSTOR: seasonal forecast and crop models in Africa.

AGRIS: “seasonal forecast” AND “crop models” in “Africa.”

Use of a range of different key words and Boolean symbols was important as search engines and portals are sensitive to the order of search key words and Boolean symbols. The technique has been found to be effective and exhaustive in searching for articles in various databases and portals [29].

**Figure 1:**
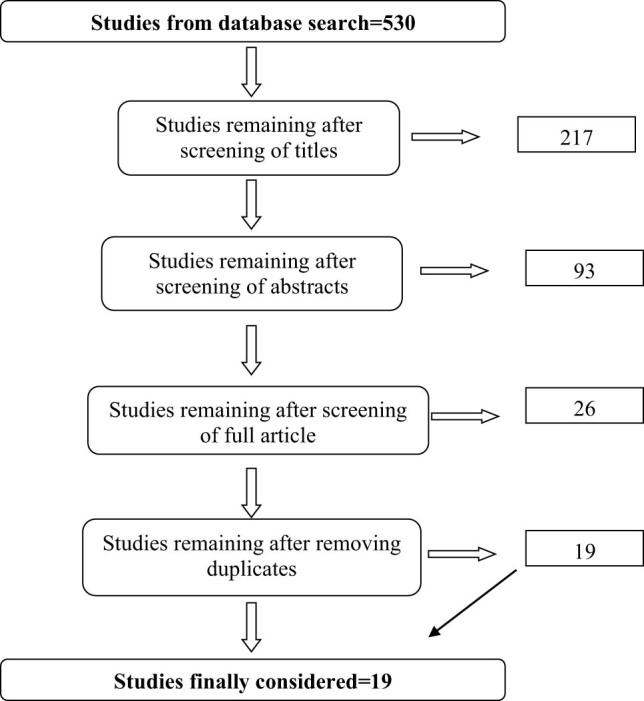
Thematic presentation of the systematic literature review process.

The initial database search yielded 530 articles across all the four databases (Figure 1):

JSTOR: 314;

Google Scholar: 132;

Web of Science: 52; and

AGRIS: 32.

The review process excluded articles, books, and book chapters that (1) are not based on the African context, (2) do not have an explicit methodology that couple seasonal forecasts and crop models for repeatability and (3) do not provide a quantitative analysis of yield responses, and (4) were not carried out over the selected period (2007–2022). The period was considered after 2007 as a new generation of models generating seasonal forecasts came into operation hence the specific period. The articles were then screened through elimination steps by assessing suitability for the study based on title, abstract, and the full reading of the article. Duplicates were removed in the process, so the study finally retained 19 articles after the process (Figure 1). Whilst this number is low, it is a considerate representation of the limited studies, which have been carried out on the continent on the subject, and this number was considered sufficient to review the scope, tools, and approaches used in Africa. Some review studies have used articles as few as six [30]. References outside the range and scope of the study were included to enable understanding of concepts despite being published outside the spatial and temporal boundaries of this study.

### 2.3 Seasonal forecasting

*Seasonal forecasts* are predictions of the average seasonal conditions across a region and over months ahead due to slowly changing parts of the climate system, e.g., about 3 months. Such predictions are difficult except under *El Niño* events, where accuracy levels are relatively high. They are often presented as tercile probabilities. The major determinant of empirical forecasts is the interaction between sea surface temperatures and atmospheric conditions. Also, seasonal forecasts use climate models to predict the size and magnitude of weather parameters. Sub-seasonal forecasts are weather predictions from 2 weeks to about 3 months. Seasonal forecasts are divided into statistical and dynamic forecasts [31].

*Statistical forecasts* are hinged on the mathematical relationship between historical, current, or expected values of predictor and the predictand. Statistical models can be grouped into analogues and stochastic disaggregation methods. Regression approach is the most common statistical forecasting technique [32]. The skill of statistically generated seasonal forecasts is relatively higher under the *El Niño* phase of the el niño-southern oscillation (ENSO) compared to the *La Niña* or *neutral* conditions. The ENSO-*neutral* phase is characterised by non-significant increase in sea surface temperatures (SST), which are not easily detected compared to usually more extreme warming and cooling characterising *El Niño* and *La Niña* seasons, respectively [32,33]. In circumstances where it is possible to account for all the predictors, statistical models require rigorous testing to ensure operational reliability. This can be improved through addition of atmospheric predictors in the statistical model [34].

*Historical analogue-based* forecasts are based on categorising historical climate predictors and identifying the future climate predictors class within historical categories. This approach is suitable when historical data are sufficiently available. Limited historical weather data reduce sample size, and the categories will not be well defined, thus compromising the methodology and forecast quality. Where there is high confidence in the predictor values resembling a specific historical season, the probability-weighted historic analogue approach is preferred. Probability-weighted historic analogue-based forecasts combine the analogue and regression approach [35]. To further improve the accuracy and efficiency of the analogue approach, the analogue can be combined with the GCM approach [36].

*Stochastic disaggregation*: Forecasts are often issued in the form of temporal summaries. To connect this information to mechanistic crop models, forecast summaries can be disaggregated into daily weather data. Stochastic weather generators create a series of synthetic daily weather data with statistical characteristics similar to the expected climate. Stochastic disaggregation captures the high frequency variability of specific weather parameters whilst reproducing the low frequency of highly variable weather events. This can be undertaken through (1) calibration of a stochastic weather generator or (2) restriction of the simulated daily weather data parameters to those of the expected forecast [37].

*Dynamic forecasting* utilises global climate models (GCMs) and regional climate models (RCMs) that mimic the land–ocean–atmosphere systems to predict weather. Notable examples include climate forecast system version 2 (CFSv2) [38], SEAS5 [39], hadley centre global environmental model [40], European centre for medium-range weather forecasts, Hamburg (ECHAM) [41]. and geophysical fluid dynamics laboratory [42]. Dynamic forecasting accounts for a wide range of land, sea, and atmospheric variables; thus, there is greater confidence in the predictions compared to statistical climate forecasting [43,44]. GCM-based forecasts are relatively more accurate at large scale (250 and 600 km) but have poor resolution at smaller scale (10 km) [45]. Parameterisation of ocean and atmospheric thermodynamics is however complex and demands more computational resources to account for numerous parameters, such as temperature and pressure [46].

*Consensus forecasts* are forecasts, which result from merger or integration of two or more forecast outputs producing a single representative forecast. Most operational forecasts especially those officially issued by governments in SSA are based on consensus from two or more forecasts. These are developed in regional climate outlook forums, where representatives of national meteorological organisations synthesise a wide variety of forecast information (local statistical forecasts, analogue analysis, dynamic forecasts, and local experience) to agree on a forecast for the season for temperature and rainfall conditions [31].

### 2.4 Crop models

*Statistical models* use mathematical relationship between predictor values such as climate summaries and outcomes of interest such as crop yield. Similarly, they produce outputs at a coarse summary scale, e.g., monthly or 3-monthly time step [47]. The reduced data requirements for model set up and simulation are an incentive to use statistical models, but this also limits assessment of some important aspects such as crop management (crop variety, irrigation, and mulching) [48]. Statistical crop models have limited capability to simulate plant phenology, water balance, or pest dynamics. They are relatively easier to calibrate but the set relationships become highly arguable under conditions, which they were not parameterised for ref. [49].

*Ricardian model* involves coupling statistical crop models with additional tools such as socio-economic models allowing for reporting crop yield changes in economic terms, which improve their usefulness in climate change and variability impact management [50]. Net farm revenues are regressed on independent variables affecting crop production, such as market price, input costs, market access, water flow, rainfall, and temperature [51]. The approach assumes that farm management decisions in climate change and variability are based on the profitability of the strategy. Decision-making in smallholder farming systems is however based on many socio-economic and bio-physical aspects some of which cannot be accounted for by Ricardian models [52]. Land valuation is challenging in smallholder farming systems of Southern Africa since most of the land is state owned; hence, there may be inconsistences. The Ricardian approach assumes that land value is indirectly derived from commodity prices. Use of the approach in Africa is consequently further limited by unregulated and weak land markets.

*Mechanistic models* determine crop productivity through mathematical relationships between plant physiological processes (e.g., photosynthesis, transpiration) and environmental conditions (e.g., soil, climate). Mechanistic crop models mimic plant phenological and physiological processes [53]. Complex cropping systems can be modelled, and the reliability increases with the availability of high-quality experimental data for calibration. Many mechanistic crop models simulate multiple crop management aspects, such as crop rotation, intercropping, cropping calendar, different crop types and varieties, fertility, irrigation, mulching, or tillage. The complexity of mechanistic models requires extensive data sets for parameterisation and calibration, which occurs inconsistently with African agricultural systems due to limited research skill or financial resources [20,27].

## 3 Results

### 3.1 Integrating seasonal forecasts into crop models

#### 3.1.1 Evolution of studies

The frequency of publications involving seasonal forecasts and crop models in Africa gradually increases over time. At some point, however, the pattern of the frequency of publications fluctuates irregularly due to a drop in the frequency of publications in 2016. The highest number of publications on the subject in question was recorded in year 2015. In years 2012, 2019, and 2021, no studies were identified that were related to integration of seasonal forecasts into crop models from our search (Table 1).

**Table 1:** Overview of the content of the reviewed studies on integration of seasonal forecasts into crop models in Africa

Author(s)	Geographical distribution	Approaches of linking seasonal forecast and crop models	Seasonal forecast sources	Crop model used	Crops covered
Mishra et al. (2008)	Burkina Faso, West Africa	Regression; stochastic disaggregation	ECHAM	System of agro-climatological regional risk analysis version (SARRA-H)	Sorghum
Hansen et al. (2009)	Kenya, East Africa	Hindcast	ECHAM	Agricultural production systems simulator (APSIM)	Maize
Sultan et al. (2010)	Senegal, West Africa	Hindcast; statistical approaches	European centre for medium-range weather forecasts (ECMWF)	General algebraic modelling system (GAMS)	Maize, peanut, maize, millet, sorghum
Zinyengere et al. (2011)	Zimbabwe, Southern Africa	Historical analogue	ENSO	AQUACROP	Maize
Gommes (2013)	Zimbabwe, Southern Africa	Hindcast	National oceanic and atmosphericadministration (NOAA)13-WINDISP14	Decision support system for agrotechnology transfer (DSSAT); World food studies simulation model (WOFOST)	Maize, wheat
Garcia-Carreras et al. (2015)	Ghana, West Africa	Statistical downscaling	ECMWF	General large area model (GLAM)	Groundnuts
Muswera (2015)	Zimbabwe, Southern Africa	Hindcast	ECMWF	DSSAT	Maize
Ramarohetra et al. (2015)	Niger and Benin, West Africa	GCM;	African monsoon multidisciplinary analysis-CATCH	SARRA-H; Erosion/productivity impact calculator	Maize, pearl millet
Paeth et al. (2016)	Benin, West Africa	Statistical yield prediction	Regional model (REMO)	model output statistics (MOS)	Peanuts, cotton, beans, yams, maize, manioc, rice, sorghum
Roudier et al. (2016)	Niger, West Africa	Hindcast	National centre for meteorological research (CNRM)	SARRA-H	Millet
MacCarthy et al. (2017)	Ghana, West Africa	Hindcast	ENSO	DSSAT	Maize
Takale (2017)	Ethiopia, East Africa	Bias correction; statistical downscaling	CFSv2	DSSAT	Maize
Asfaw et al. (2018)	Ghana, West Africa	Hindcast	WATCH forcing data methodology applied to ERA-Interim data	GLAM	Maize
Ogutu et al. (2018)	Kenya, Ethiopia, Tanzania, East Africa	Probabilistic prediction	ECMWF	WOFOST	Maize
Roudier et al. (2011)	Niger, West Africa	Hindcast	PRESAO	SARRA-H	Millet
Laudien et al. (2020)	Tanzania, East Africa	GCM	GCM (climate hazards group infra-red precipitation with station data)	Machine learning LASSO regression	Maize
Badmus and Ariyo (2011)	Nigeria, West Africa	Hindcast		Autoregressive integrated moving average (ARIMA)	Maize
Malherbe et al. (2014)	South Africa, Southern Africa	Hindcast	GCM ECHAM 4.6	Model outputs statistics	Maize
Oettli et al. (2011)	Senegal, West Africa	GCM	RCM (HIRHAM, CLM, HadRM3P, RegCM, RACMO, REMO, RCA, PROMES, IRAJ)	SARRA-H	Sorghum

#### 3.1.2 Spatial distribution of studies

**Figure 2:**
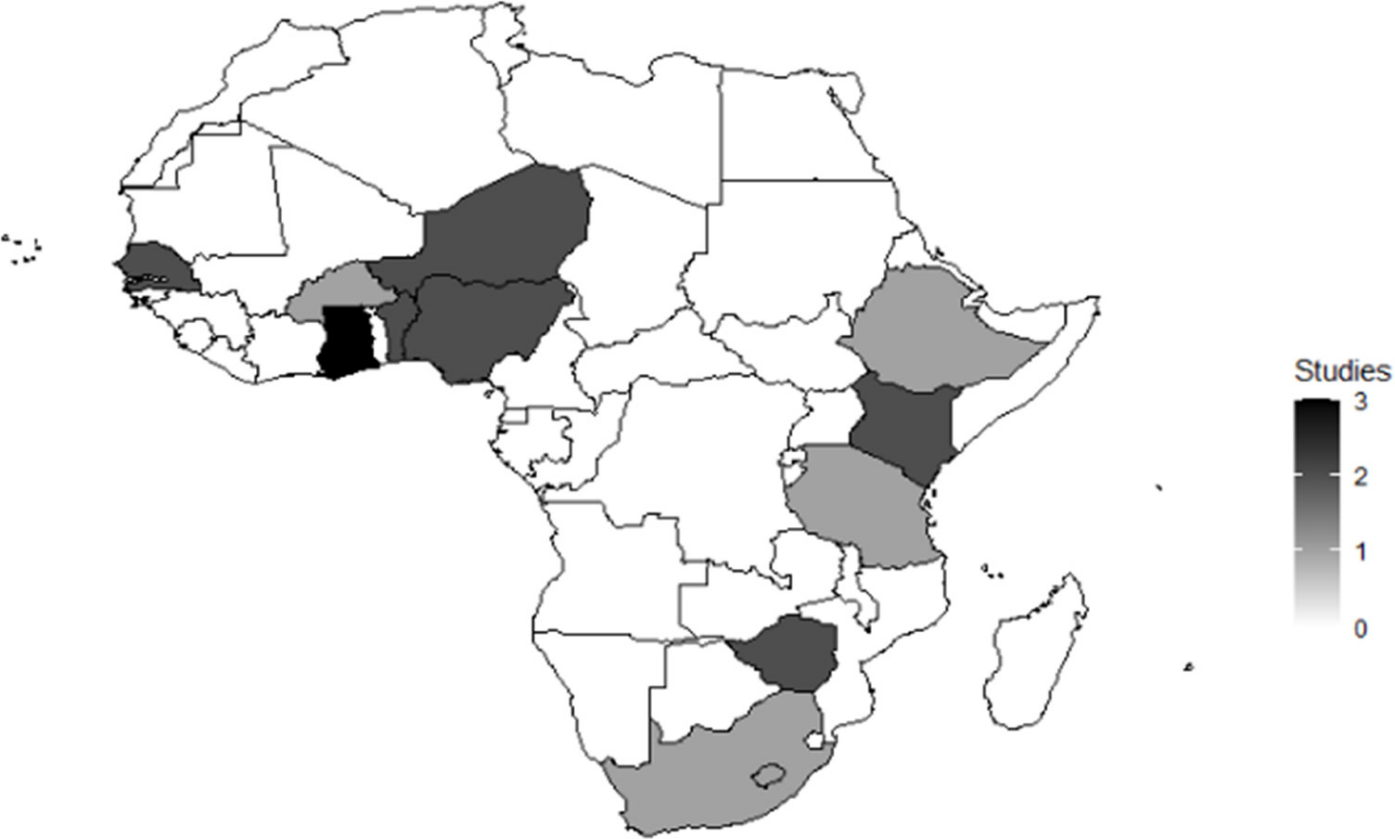
Spatial distribution of studies on integration of seasonal forecasts and crop models in smallholder farming systems in Africa for the period, 2007–2022.

Almost the entire African continent has been covered by research involving different aspects of integration of crop model and seasonal forecasts. Table 1 and Figure 2 provide a general overview of the reviewed studies in terms of geographical distribution, sources of seasonal forecast information, crop models, and techniques used to integrate seasonal forecast and crop models in the region (Table 1, Figure 2). Most of the studies are concentrated in west Africa (58%) (11). Most of these were in the arid to semi-arid Sahel region. Limited research was undertaken in eastern and southern Africa in proportions of 16% (4) and 18% (3), respectively. No studies were undertaken in central and north Africa during the period under consideration (Table 1 and Figure 2).

#### 3.1.3 Crops covered

Studies that integrate seasonal forecasts and crop models covered a wide range of crops. Maize, a common staple food crop for Southern Africa and parts of East Africa, was the most common crop across all the studies in Africa (Table 1). Most of the crops covered by studies on the integration of seasonal forecast information and crop models were cereals (78%) and were distributed in all of Africa except north Africa. Maize crop was generally distributed across all regions of Africa except North Africa. Drought-tolerant crops such as sorghum or pearl millet were also the subject of research (27%), largely in the west African region but with no research being undertaken in other parts of Africa. In addition, there was limited research involving peanuts (11%) and it was only limited to west Africa. All the other crops were represented in very low proportions. Beans, cotton, groundnuts, manioc, rice, wheat, and yams are also studied in a few instances, predominantly in West Africa (Table 1) [54,55].

#### 3.1.4 Application of research outputs

Application of research outputs is strategic, tactical and operational representing long-term and inter- and intra-seasonal temporal spans [56]. At least 60% (11) of the studies were utilised for tactical and operational decision-making with [57] being a notable example [54], focused on tactical decision-making only, for example, improved cropping technology such as hybrid seeds compared to current and traditional seeds. At least 74% (14) of the studies focused on yield prediction, but it was either under tactical or operational decision-making not just sole yield prediction under seasonal forecast information scenarios with [54,58] being the most notable. Only two studies [59,60] interpreted results in a policy discussion towards enhanced food security (Table 2).

**Table 2:** Application of research integrating seasonal forecast information into crop models

Author(s)	Yield projection	Operational decision	Seasonal decision support	Policy
Mishra et al. (2008)	✓	✓	✓	
Ogutu et al. (2018)		✓	✓	
Sultan et al. (2010)		✓	✓	
MacCarthy et al. (2017)	✓	✓	✓	
Asfaw et al. (2018)	✓	✓	✓	
Muswera (2015)			✓	
Ramarohetra et al. (2015)	✓		✓	
Takale (2017)	✓	✓	✓	
Gommes (2013)	✓	✓	✓	
Garcia-Carreras et al. (2015)		✓	✓	
Paeth et al. (2016)	✓		✓	✓
Roudier et al. (2016)		✓		
Zinyengere et al. (2011)	✓	✓	✓	
Hansen et al. (2009)	✓	✓	✓	
Roudier et al. (2011)	✓		✓	
Laudien et al. (2020)	✓		✓	
Badmus and Ariyo (2011)	✓		✓	✓
Malherbe et al. (2014)	✓			
Oettli et al. (2011)	✓			

#### 3.1.5 Farm management practices

**Figure 3:**
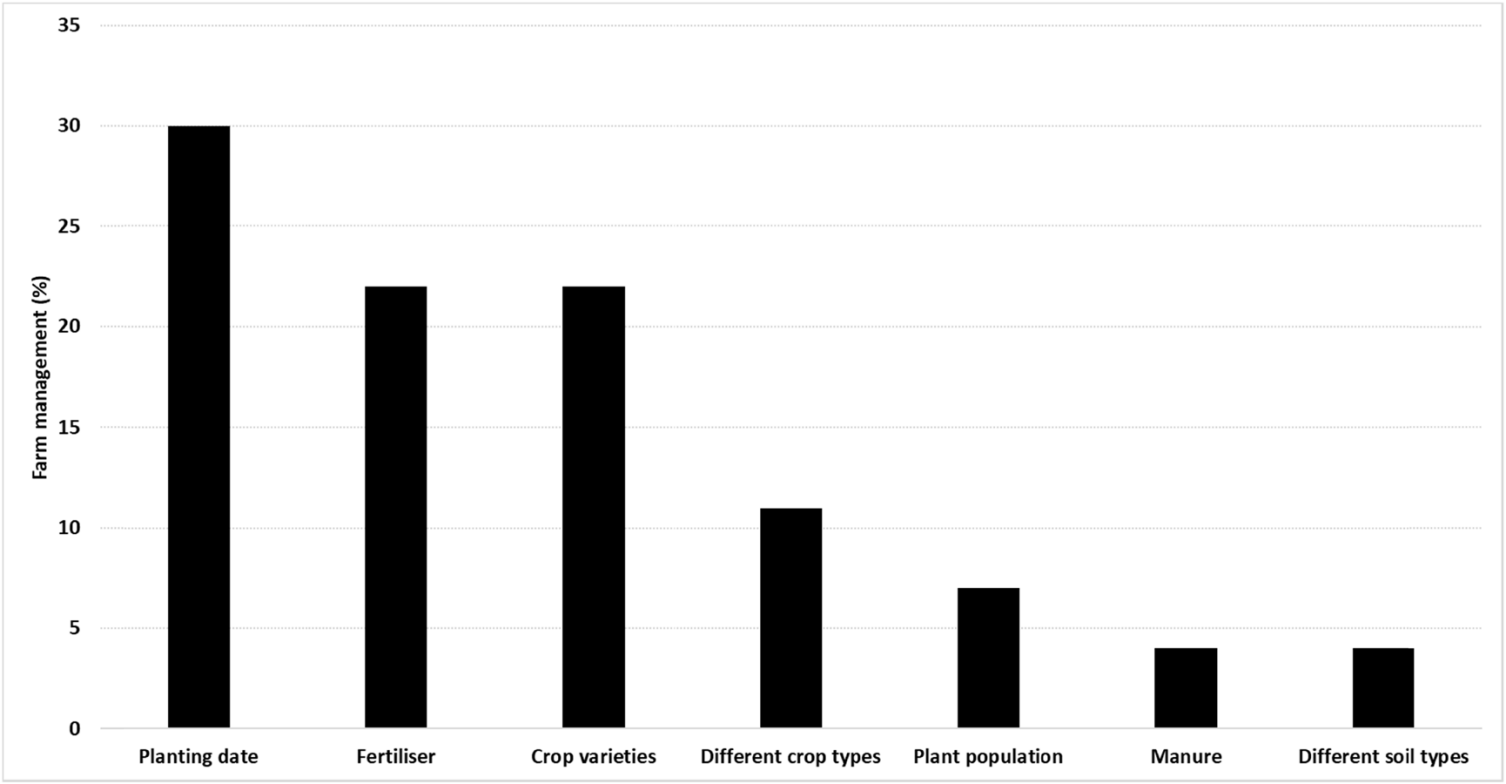
Range of farm management practices assessed using integrated seasonal forecast information and crop models.

Most studies (58%) in the region involved assessment of farm management practices using seasonal forecast and crop models. There was a higher density of farm management practices in west Africa as most of the studies were based in the sub-region (Table 2 and Figure 3). Planting dates were the most common practice in west Africa. They were also less distributed in other regions such as east and southern Africa. Similarly, fertiliser use was more dominant in west Africa compared to other regions. Change in choice of cultivar was evenly distributed across all regions except regions where no research on integrating seasonal forecast and crop models was undertaken (Figure 3).

Most of these studies (Figure 3) assessed the impacts of a range of planting dates on crop yields given certain specific weather information [61]. Some studies assessed single farm management practice [62] whereas others assessed a combination of management practices [63,64]. A notable proportion of studies assessed productivity from different crop types, such as maize, peanut, millet, and sorghum [55], as shown in Figure 3, which also included the assessment of different varieties, such as early and late maturing maize varieties [62]. Some studies evaluated fertility through organic and inorganic fertilisers, such as manure under seasonal forecasts [63]. A few studies only assessed shifts in cropping locations to manage climate risk and this involved evaluating the changes in soil type in the different agro-ecologies [64] as seen in Figure 3.

### 3.2 Tools for integrating seasonal forecasts and crop models in Africa

#### 3.2.1 Seasonal forecast information

The sources of seasonal forecast information used in past studies were diverse. At least 70% of the studies derived forecasts from GCMs, with European Centre for Medium-Range Weather Forecasts (ECMWF) and ECHAM being the most common. About 11% of the studies were related to the ENSO cycle (Table 1), which is a major climate driver over Africa [65,66] (Table 1).

Most of the forecasts across the studies were derived from the ECMWF (System 4) model (Table 1), which is developed and run by European-based forecasters [67]. Most of the research in Africa was spearheaded by European-based researchers who were also working with the ECMWF model, hence the increased frequency of use of the model. Only about 18% (3) of such studies used the ECHAM model. ECHAM is a GCM developed by the Max Planck Institute for Meteorology [43,54,68]. Some other sources of seasonal forecast data included the CFSv2 and CNRM GCMs, which were used in about 12% (2) of the studies. CSFv2 is a coupled ocean‐atmosphere‐land model that was developed by the National Centers for Environmental Prediction (National centre for environmental prediction/NOAA) [69] whilst CNRM was developed by the National Centre for Meteorological Research [70]. About 6% (1) of the studies used Climate Hazards Group Infra-Red Precipitation with Station, which is satellite data aggregated and maintained by the United States Geological Survey and Climate Hazards Center [71]. About 12% (2) of the studies used RCM as a source of seasonal forecast information [72]. One of the studies evaluates nine RCMs (HIRHAM, CLM, HadRM3P, RegCM, RACMO, REMO, RCA, PROMES, and IRAJ) for accuracy and feasibility in predicting crop yields [73]. Many of the above studies also explicitly considered ENSO conditions (i.e., *El Niño*, *neutral*, and *La Niña*) as driver of the produced seasonal forecasts [63] (Table 1).

#### 3.2.2 Crop models

The reviewed studies show a wide range of crop models being utilised in research involving the integration of crop models and seasonal forecasts. At least 74% (14) of the studies used mechanistic models whilst 16% (3) used statistical models and the other 5% (1) also used the Ricardian approach (Table 1).

The most widely used mechanistic models in Africa were Decision Support System for Agrotechnology Transfer (DSSAT) [20], SARRA-H [74], GLAM [61], WOFOST [75], and APSIM [27] in about 21% (4), 21% (4), 12% (2), 6% (1), and 6% (1), respectively (Table 1). Both APSIM and DSSAT models have been widely used across the continent, mostly in Southern Africa, and to a lesser extent in East Africa [43]. The SARRA-H model is mostly widely used in west Africa as it is effective in simulating crop yields under tropical conditions prevalent in the region [64]. WOFOST is a single location model effective under homogeneous conditions, which has been recently adopted for regional-based simulations, for instance in East Africa [75]. GLAM is a statistical crop model well suited for large area assessments of regional agricultural yields. Statistical models include model output statistics, such as used by ref. [60] to simulate crop yields under climate variability in West Africa. The Ricardian approach was utilised in the form of GAMS, a bio-economic model that was utilised to assess the economic benefits of farmer’s decision-making given priori seasonal forecast information. Some statistical models such as ARIMA were used in a single study [59] as indicated in Table 1.

### 3.3 Integrating seasonal forecast and crop models in Africa

#### 3.3.1 Approaches for linking seasonal forecast and crop models

The study identified three techniques that have been utilised to integrate seasonal forecasts and crop models (Table 1). About 83% of the integration techniques are based on historical analogues with a notable proportion based on GCM data conditioned on historical SST and other atmospheric conditions. A notable proportion (20%) of analogue techniques were based on the ENSO phases. An example of the research by ref. [63], where seasonal forecast information was derived from hindcast use of ENSO-based phases, integrated with the DSSAT model to assess optimum sowing dates when subjected to different crop management practices. About 11% of the integration techniques were directly fed into the crop model without prior conditioning. This involved extraction of seasonal forecast data for a specific location. For instance, ref. [61] used the GCM-based ECMWF directly with large area models, such as GLAM. About 5% of the techniques used the statistical yield prediction technique and these however only utilised RCMs as the source of seasonal forecast data. This involved the use of REMO, an RCM [60], as shown in Table 1.

##### 3.3.1.1 GCMs

GCM output from the ECHAM v4.5 has been used as inputs to the SARRA-H crop model for sorghum yield prediction under a range of crop management practices and seasonal forecasts in West Africa [54]. Similarly, ref. [76] used CFSv2 forecasts to predict maize for food security for planning purposes in Ethiopia, using the DSSAT crop model. In this approach, GCM data are conditioned based on statistical parameters of the historical measured data.

Statistical yield prediction was undertaken in about 6% (1) of the studies. For instance, this was utilised by ref. [60] where MOS, a statistical model, was coupled with REMO, an RCM to evaluate the sensitivity of various crops (e.g., peanuts, yam, maize, millet, rice, soybeans, and sorghum) to climate change (Table 1).

### 3.4 Changes in productivity due to the use of integrated seasonal forecast and crop models

**Figure 4:**
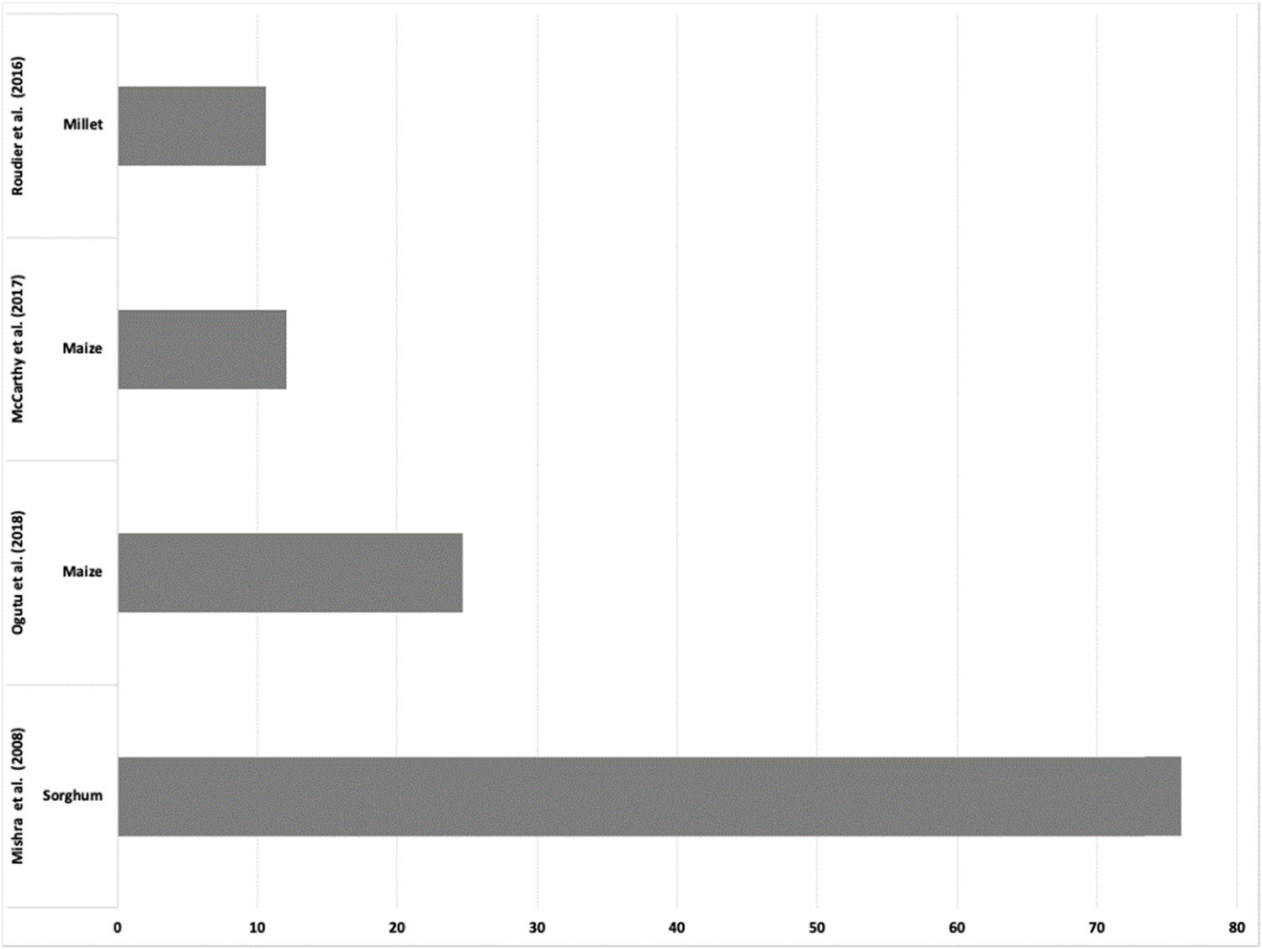
Projected changes in crop yields from seasonal forecast information integrated with crop models in Africa (2007–2022).

Yield changes from seasonal forecasts and crop models were consolidated in horizontal histograms. Some studies did not provide the actual numeric changes but rather showed them in spatial format; hence, fewer studies were used in the analysis. Decision-making under seasonal forecast led to the highest crop yield increase of 76% for sorghum. Similarly, maize also had notable increases in yields (12–24%) given seasonal forecast information. Millet also responded favourably to decision-making under seasonal forecast with the yield increases of 11% (Figure 4).

## 4 Discussion

### 4.1 Use of crop models for assessing climate variability management

One of the main aims of the study was to identify and critique the tools used to integrate seasonal forecasting and crop models. With regard to crop models, most of them require calibration with historical data prior to yield prediction [20,27]. The accuracy of simulations is highly dependent on proper calibration, which in turn hinges on the quantity and quality of calibration data. Statistical crop models do not need detailed calibration such as in mechanistic crop models, as they require fewer input data such as historical weather data (rainfall and temperature) and crop yields for model parameterisation and calibration. Statistical crop models may be more suitable to African agricultural research where there is limited data collection due to limited research skill and financial constraints, to support agricultural research [77]. Statistical models can therefore be used in yield simulations as they simulate yields with limited data for calibration [78]. Statistical models are mostly limited for use in yield prediction. They are based on the direct relationship between climate and yields. Yield prediction is therefore of limited accuracy as they do not account for most of the factors that determine crop growth and development.

Statistical crop models account for seasonal forecasts as climate summaries to determine the relationships between climate and crop. They are therefore compatible with seasonal forecast information, which is mostly issued as temporal and spatial summaries. They however cannot be used for predicting location-specific crop yields due to the coarse spatial resolution [37]. Mechanistic models are not directly compatible with some seasonal forecast information, which is issued out in temporal and spatial summary formats [24]. Advances in climate science have improved seasonal forecasting and their processing. Seasonal forecast data need to be downscaled to a daily time step to be usable by mechanistic crop models. Statistical and dynamic downscaling are the most common downscaling techniques. Downscaling however reduces the accuracy of forecasts compared to forecasts at the summary scale [79]. Processing of seasonal forecasts can lead to disaggregation of the forecast summaries to a daily time step (Section 3.2) [35]. Such information can therefore be linked with process-based models requiring weather input at a daily time step. The accuracy of such disaggregated forecast data is however limited [25]. Despite such challenges, the study realised that use of statistical models and the Ricardian approach still lags behind mechanistic models (Table 1). This is attributed to the fact that most of the research integrating crop models and seasonal forecasts evaluates farm management practices and climate variability options (Table 1). Mechanistic crop models have higher accuracy in the simulation of crop growth and development as well as simulation of crop management aspects compared to statistical models.

Despite the huge data requirements, mechanistic models, especially DSSAT and APSIM, are still the most widely used models in the region. This is mostly attributed to pre-project commencement bias, where models for use in certain specific projects are decided based on the project members’ experience and knowledge of such models. There is greater support on models such as DSSAT, where organisations such as the DSSAT foundation regularly undertake trainings in Africa. Most Australian centre for international agricultural research-funded projects involve a modelling component and they also provide the necessary training and support on the use of models such as APSIM [80,81]. The cycle therefore continues where beneficiaries of such projects and training will also use similar models in future research; hence, the use of such models (DSSAT and APSIM)) is predominant [82]. There is limited capacity building and support involving some models such as AQUACROP, despite the tool requiring less data and less calibration efforts than APSIM or DSSAT crop models.

Mechanistic models allow simulations of conditions that are not restricted to historical conditions. They can therefore reliably simulate crop yields under projected climate variability conditions. Mechanistic models utilise a wide range of data for parameterisation and calibration. This also includes different aspects of weather. This allows for yield predictions under non-previously experienced weather phenomena. Such models can therefore be utilised in climate variability adaptation research [25]. On the contrary, statistical and Ricardian models cannot account for climate variability with high confidence. Statistical models are mostly based on the direct mathematical relationship between yield and yield determinants. The direct relationship therefore makes it difficult to account for other changes introduced under climate change. Ricardian models do not account for other parameters as they are mainly based on the land value. Mechanistic models are therefore the only type of models that can account for climate variability.

Mechanistic models also mimic the normal plant bio-physical and physiological processes, such as photosynthesis and respiration. They can thus accurately be used to simulate different aspects of crop growth and development under climate variability. Mechanistic models also account for crop management aspects, such as mulch, variety, fertiliser, tillage, and soil information. These are also some of the management practices altered by farmers in climate variability adaptation [20,27]. They cannot account for daily farm management practices, such as mulching, fertiliser application, and irrigation; hence, their use is limited in the region. Their use is likely to be reduced in the future as climate change adaptation research increases. There is an increased need for the assessment of climate variability adaptation in the region, which increases the need for the use of mechanistic crop models compared to statistical models.

The Ricardian approach is advantageous in regions or countries with functional land markets. In Africa, use of this approach is limited by unregulated and weak land markets. Land valuation is a challenge in smallholder farming systems in Africa since most of the land is state owned; hence, there may be inconsistences in pricing. The prices attached to the commodities are constant whereas in reality, prices fluctuate, leading to under- and over-estimation of gains and losses, respectively. The approach has limited use in the region as it mostly dwells on the economic impacts of a range of farm management decisions. The approach can potentially be more useful in the future where there is an increasing need for assessing the economic impacts of climate change. The model however relates to statistical models, as there is limited flexibility in accounting for farm management decisions. Some models such as CLEM can therefore be used as they account for farm management practices as well as economic impacts.

### 4.2 Linking seasonal forecast information to crop models

There is a need to identify and critique the range of approaches used to link crop models and seasonal forecasts. This is important to identify the most ideal approaches in different conditions for agricultural research in Africa. The review realised three major techniques that can be utilised to integrate seasonal forecast information into crop models. These are statistical yield prediction, analogue approach, and use of GCM outputs. The techniques are used to produce seasonal forecasts in a format suitable for linking seasonal forecast with models. Process-based models, which are the most appropriate for use in climate change adaptation research, require input forecast data at a daily time step [20,27]. Hence, there is need for the assessment of the most appropriate seasonal forecast production technique, whose forecast output is more compatible with the input requirement of process-based crop models.

The analogue-based seasonal forecast generation approach is built on historical weather data whose parameters are similar to those of the forecasted conditions for specific localities. The approach therefore makes it easier to couple seasonal forecasts and process-based crop models. In addition, the analogue approach is advantageous when utilised at the spatial and temporal scales at which the historical weather data are available [35]. The approach requires high-quality and quantity climate data. In Africa, challenges in skill, financial resources, and management of weather data collection pose complications for obtaining the requisite climate data. Furthermore, weather data collection is dominant and of highest quality and quantity in urban areas, research sites, and locations of special interest. These areas are not always where the smallholder farming systems are dominant. This could be the reason why a lot of the studies are mostly for research purposes and not directly focussed on application in smallholder farming systems or policy. Increased climate variability reduces the confidence in the analogue approach, as anthropogenic factors influence immediate future weather. There is greater confidence in the use of historical analogues when the season under consideration is characterised by higher probability of the occurrence of phenomena such as *La Niña* or *El Niño* [83].

The GCM-based seasonal forecasting generation approach involves direct linking of GCM output data and crop models with limited processing. This reduces the need for technical expertise and errors in converting forecasts into the daily weather format compatible with crop models. The GCM output data may need further processing to improve accuracy [35]. The statistical yield prediction approach has limited compatibility with seasonal forecasts as it predicts crop yields based on predictor variables through repeated conditioning of the crop model yield outputs. This minimises technical demands as well as compounding of errors associated with downscaling seasonal forecasts into the daily weather format and interaction with process-based crop models. The GCM approach produces relatively credible crop yield forecasts, although accuracy can be improved. Despite the compatibility with crop models, the coarse resolution associated with GCM outputs presents challenges leading to the prediction of erroneous yields, which do not account for local climatic variations. Accounting for local climatic variations is important for capturing location-specific climate risk. Advances in atmospheric science have increased spatial resolution of GCM outputs, but there are chances of distorting daily rainfall. There is therefore need for further attempts to minimise the yield prediction bias to effectively apply this approach [25].

With regard to statistical yield prediction, the yield outputs are not very accurate as the process is not representative of normal crop growth and development. The approach assumes a direct linear relationship between the predictor and crop yields, which is not characteristic of normal crop growth and development. Such an approach cannot account for in-season changes in weather and management; hence, it may not be appropriate for the assessment of performance of farming practices given specific seasonal forecast information.

With regard to stochastic disaggregation, especially the parametric approach, there is greater confidence in the daily sequence outputs since they are based on historical weather patterns. The approaches, however, cannot produce out-of-parameterised events such as non-previously experienced extreme rainfall, temperature, dry, and heat spell [84]. However, there are however greater chances of predicting parameters of extreme variability (e.g., extreme rainfall and temperatures) using the non-parametric based mode of the stochastic disaggregation approach, since it is not based on historical climate data [35]. This is best suited to the African context where the region is experiencing increased frequency of climate variability.

The review did not consider studies that use stochastic disaggregation in integrating seasonal forecasts and crop models. The approach has been highlighted as one of the major techniques to integrate seasonal forecasts and crop models [35], yet has not been applied by studies in Africa. This is potentially attributed to the greater technical demands as well as the heavy computing demand.

The few studies reviewed in the study are a sign of fewer studies integrating seasonal forecasts and crop models in Africa. This is in comparison to other studies, which have reviewed 19 studies [85,86] and at least 35 articles [29]. The limited research is attributed to relatively high technical demands. Capacity building through grants, workshops, and trainings may potentially improve the ability of researchers to undertake such research with limited difficulties. Such capacity building is relatively greater in West Africa as realised by the greater number of research consortia such as the West African Science Centre on Climate Change and Adapted land Use undertaking seasonal forecast-related research in the regions. This contrasts with Southern Africa and east Africa, where there are few research consortia focused on coupled seasonal forecasting and crop modelling as signified by the fewer studies in recent years. The limited studies in East Africa might be attributed to the presence of two cropping seasons. This therefore challenges accuracy of seasonal forecasts, thus potentially limiting the number of studies combining seasonal forecast information and crop modelling in this area.

### 4.3 Opportunities in integrating seasonal forecast information and crop models

In addition to the key lessons on critiquing tools and methods utilised to integrate seasonal forecasts and crop models, those like GCM-based approaches are more efficient at integrating crop models and seasonal forecasts. The review identified key lessons and opportunities and additional lessons. These are as follows: (1) there are very few studies undertaken in Africa to inform decision-making based on integrated seasonal forecasts and crop modelling; (2) few crops are covered with maize and other staples; (3) integrated modelling is mostly used to inform yield prediction and operational decision-making with limited studies being on policy; and (4) the predominant farm management practices evaluated in studies on integrating seasonal forecast and crop models were planting date, fertiliser, and crop varieties.

Despite notable research integrating seasonal forecasts and crop models, application of seasonal forecast information in smallholder agriculture in Africa is limited. The application is limited to the provision of weather information to enhance operational decision-making. To widen research into the use of seasonal forecasts and crop models, further opportunities exist for enhancing the quality of outputs from integrating seasonal forecasts and crop models.

Most of the studies focused on the maize crop since it is a staple food crop for most households in Africa [87]. The crop is also a source of raw materials for the feed and many agro-related industries [88]. It is therefore important to assess the response of such a crop to seasonal forecast predictions as well as develop tools for sustainable production under climate change. Focus on the maize crop might be attributed to the increased body of knowledge on model calibration of the crop as most of the research on crops in Africa is biased towards maize [89,90,91]. This also highlights the need for research on other crops of economic interest and neglected crops such as bambara nut and mung bean [92]. Cassava has not been the focus of such studies, but it is one of the most important crops for food, feed, and industrial use on the continent. Drought-tolerant crops, such as sorghum, millet, and cowpeas, that could minimise the impacts of climate variability can also be the subject of research given the increased climate risk.

The review realised that there is a “blindspot” in the regions and countries undertaking research on integrated seasonal forecast and crop models to enhance decision-making in Africa. Such research has not been undertaken in North, Central, and Western Southern Africa. There is therefore need to undertake such research in these regions to enhance farmer decision-making. Such efforts also come with other benefits such as improved skill on seasonal forecasts in these areas. The other reason for the limited studies of seasonal forecasts and crop models in central Africa is attributed to the limited climate risk due to predominantly high rainfall. The value of investing in forecasts is therefore relatively low. On the contrary, Western Southern Africa and North Africa have high climate risk due to predominantly high temperatures and low rainfall. It is therefore needful for investment in research on seasonal forecast and crop models, as this has a higher value to farmers in the region.

Future research involving seasonal forecast information in Africa can utilise seasonal forecast downscaling techniques. Spatial downscaling allows for extrapolation of location-specific seasonal forecast data. Recommendation and decision-making based on seasonal forecast from specific sites can therefore have more value compared to forecasts at greater spatial scale. The use of downscaled data can improve the effectiveness of the corresponding recommendations. Such downscaling techniques can be either statistical or dynamical [93]. Future research should also focus on areas where there are farming activities. There are however challenges associated with the limited availability of measured weather data as well as research stations in those areas. Use of local weather and atmospheric data can potentially improve the quality of the downscaled forecasts.

There are multiple types of crop and climate models that can be utilised in studies integrating seasonal forecast information and crop models. Different types of models have different strengths and weaknesses. There are a range of climate models with differences in the drivers and function. As a result, there is greater uncertainty in seasonal forecasts, which translates to uncertainty in crop yield forecasts. The accuracy and reliability of climate forecasts can be enhanced through the use of climate forecasts from multiple climate models, which can be both dynamic and statistical. This enhances accuracy and reliability through drawing from the strengths and compensating on the weaknesses from each model. This has been successfully undertaken in research under the development of a european multimodel ensemble system for seasonal to inTERannual prediction project where an operational seasonal forecasting system was designed using multiple coupled models, leading to reduced forecasting error [94]. The use of ensemble means from multiple forecasts, which have been found to be more skilful compared to outputs from single model forecasts. The use of multiple models can potentially minimise errors resulting from individual seasonal forecasting models [95]. Most of the uncertainty in seasonal forecast prediction is attributed to dynamics of initial conditions and model structure. This is managed by averaging data from different initial conditions within the same model, and model uncertainty is minimised by averaging outputs from different models [96]. The agricultural model intercomparison and improvement project initiative compared and aggregated crop model inputs and outputs [97]. Such an approach is critical in increasing the confidence in localised simulation outputs and recommendations. Crop models are unable to completely mimic crop growth and development; hence, they have different specialties. For instance, mechanistic models such as DSSAT [20] can account for daily phenological and physiological crop growth and development, whereas AQUACROP mainly focuses on soil water dynamics. Integrating and aggregating mechanistic, dynamic, deterministic, and stochastic crop models minimises errors and complements shortcomings of other models [97]. The review assessed a range of crop models (Figure 4). Integrated use of multiple crop models and different seasonal forecast information outputs from different models could improve decision-making, through the use of a wide range of model outputs, which can reduce uncertainty. Such can be useful when focusing on localised recommendations whose accuracy can be limited through downscaling, where downscaling can minimise the accuracy of projected forecasts.

Integrating seasonal forecasts and crop models adds value through providing a platform for assessing the various management decisions under projected forecasts. The review realised a limited assessment of such farm practices. This falls short of the amount and type of practices utilised by farmers in managing climate variability [98]. Farmers utilise a wide range of management practices; there is therefore need for the assessment of a wide range of practices. In addition, farmers utilise farm management practices as a combination of practices as compared to individual practices as highlighted in the review. There is therefore the need to evaluate the farm management practices as combinations of practices to mimic small holder’s farmers actual management. Evaluations can therefore be undertaken on a combined set of practices, under smallholder farmer conditions using integrated seasonal forecasts and crop models. Process-based mechanistic crop models are capable of directly and indirectly simulating combined set of practices as they can account for key farm management practices.

The review realised that integrated modelling is mostly used to inform yield prediction and operational decision-making with limited studies focusing on policy (Table 2). There is therefore the need to bridge the gap between seasonal forecasting and crop modelling with policy. Such a link would be essential in ensuring that the outcomes from research can be recommended to farmers by the government. This can potentially lead to increased use of forecast information to enhance decision-making in farming. One of the challenges of using forecasts has been the limited forecasting skill, which reduces uptake of seasonal forecasts amongst farmers. Hence, there is an increasing need for advancement in climate science to improve forecast skill [99]. Such increased confidence in forecast skill will increase the confidence of such policies by the government for use by the farmers.

Most of the research work in operational seasonal forecasting in Africa has been undertaken by researchers in universities and established research institutions. The technique of integrating seasonal forecast information and process-based crop models to enable improved farm management decision-making is not compatible with the literacy levels of most smallholder farmers. The information is however important in farmer decision-making to improve productivity under climate variability conditions. Extension services can potentially contribute to the proper communication of outputs from the scientific community to local farmers. Government agricultural extension workers interact with farmers on a regular basis and have relatively high literacy. They can thus undergo training to acquire knowledge on the theory and application of integrating seasonal forecast into crop models. They can therefore be a bridging gap between researchers and farmers, through provision of recommendations from integrating seasonal forecasts and crop models. Such an initiative enhances formulation and implementation of policy related to the use of integrated modelling in decision-making [100].

Seasonal forecast information has been beneficial to some smallholder farmers in Southern Africa specifically in the North-Western province of South Africa. During the 1997/1998 season, there was an intensive awareness campaign on the impending *El Niño* and its corresponding impacts on crops. Smallholder farmers responded through making corresponding farm management decisions (e.g., reduction in land area, increased moisture conservation, off farm activities) [101]. This proves that given seasonal forecast information, smallholder farmers can make the appropriate tactical farm management decisions. Some of these climate variability management decisions can be simulated using process-based models, such as cropping system, alternate seed varieties, water harvesting, conservation agriculture, irrigation, and nutrient efficiency [20,27].

### 4.4 Changes in crop productivity attributed to the use of seasonal forecasts and crop models

From the study, there is evidence of increased yield productivity attributed to the use of seasonal forecast-based predictions compared to the default practices (Figure 4). The increased productivity is attributed to the informed decision-making on the use of seasonal forecasts compared to default practices. For example, the use of seasonal forecasts potentially leads to the determination of sowing at optimal time, which could lead to higher yields [102]. On the contrary, currently practices are based on farmer experiences or instinct; hence, they may lead to sowing during non-optimal time periods, which may lead to poor productivity. The use of default practices thus increases the chances of sensitive crop phenology stages coinciding with unconducive conditions such as dry spells, which leads to reduction in yields. In some cases, use of such practices have proved to be valuable as farmers use long time experience and indigenous knowledge. The use of such practices are increasingly unreliable due to climate change and cultural loss. The use of seasonal forecasts and crop models therefore offers alternative forms of decision-making, as this leads to increased productivity.

The systematic literature review considered 19 studies, but 4 studies displayed actual yield figures with the rest showing spatial maps where figures cannot be extracted. This highlights the limited research on integrating seasonal forecast information and crop models in Africa. There is therefore an increasing need for such research to increase knowledge on the different approaches that can be used to predict yields and enhance decision-making under seasonal forecasting. Systematic reviews using more studies could therefore give greater confidence in the outputs of such reviews.

### 4.5 Conceptual framework

**Figure 5:**
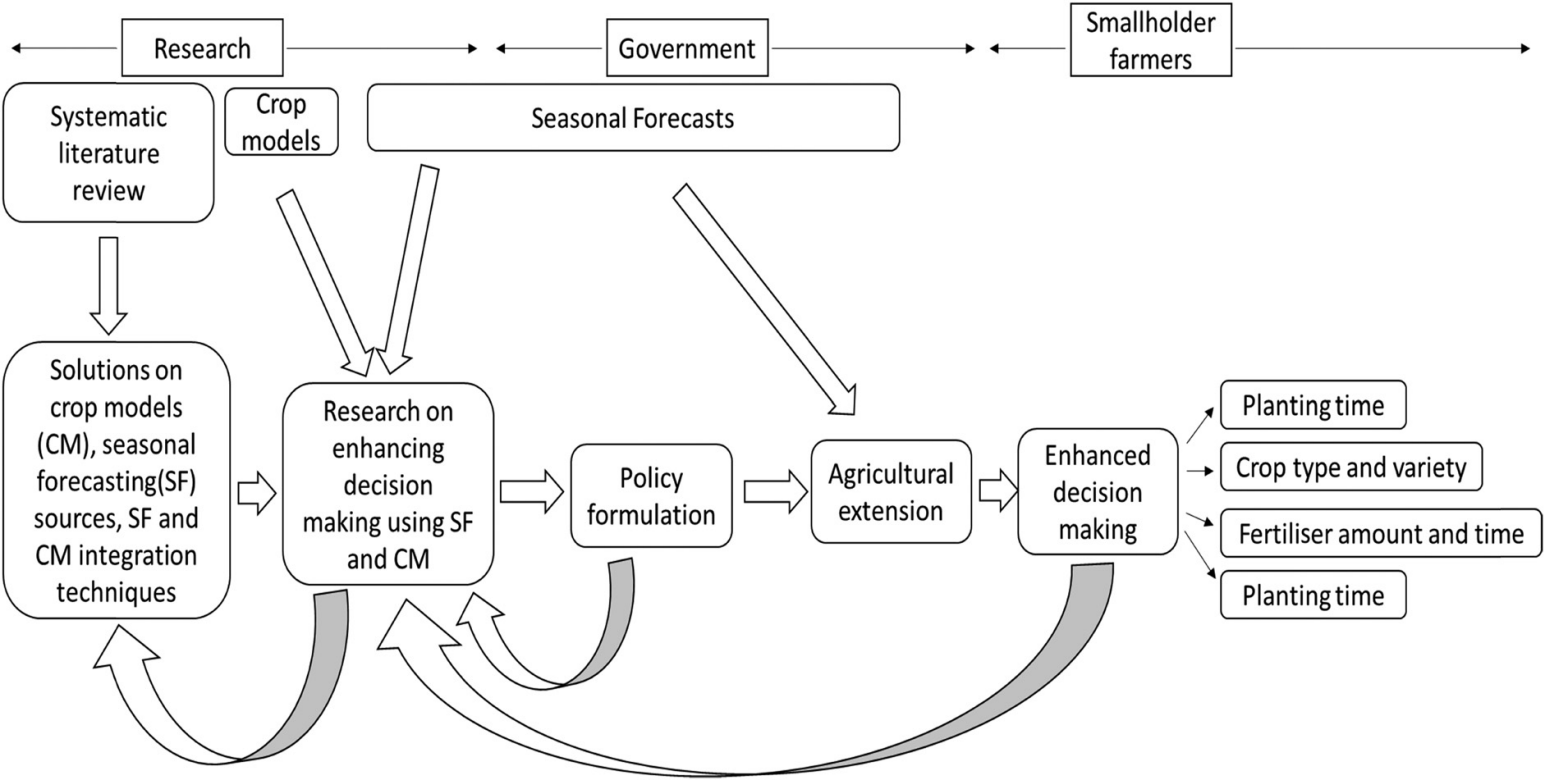
Conceptual framework highlighting the “seasonal forecast value chain,” potential value and application of integrating seasonal forecast information and crop model in Africa.

For effective and sustainable use and application of seasonal forecast information, there is need for a clearly defined pathway (Figure 5). Seasonal forecast information can be developed and disseminated by the research institutions and government meteorological agencies. In most cases, governments use consensus forecasts. These are usually broadcasted via radio, TV, and newspapers. The medium-to-long-term forecasts are usually coarse and probabilistic and are useful for strategic crop management and short-term forecasts, which are much more granular and accurate, are useful for operational decision-making (e.g., fertiliser timing).

For the effective use of seasonal forecast information, researchers can integrate forecast information with crop models. Beforehand, there is need for efforts to understand the sources of seasonal forecasts and choice of crop models. This is undertaken potentially through systematic literature review. There are various types and sources of seasonal forecast information with GCMs and historical analogue data being the most predominant. The choice of crop model needs to be determined based on the target aim of the research such as recommendation of appropriate crop management practices.

The outputs from integrating seasonal forecast and crop models can potentially be utilised by the government for policy formulation. Government extension agents partially implement the policy through disseminating the research recommendations to farmers. Such recommendations can potentially lead to enhanced decision-making through use of the optimum planting time, appropriate crop type, and variety as well as optimum fertiliser type, amount, and timing.

## 5 Conclusion

The review consolidates lessons from previous research on the tools, approaches, and application by researchers who couple seasonal forecast information with crop models, as an approach to enhance climate variability management in smallholder farming systems in Africa. The review shows that most recent work on the integration of seasonal forecast into crop models has been predominantly over West and Eastern Africa, with limited work being done in other regions of Africa. Specifically, there is need for more research in central and north and south-western Africa, where no studies have been recorded in recent times. Maize crop dominates research on the integration of seasonal forecasts and crop models, but this provides a foundation to focus research on other crops of economic interest. This also includes increased focus on drought-tolerant crops as well as widening cropping systems, with emphasis on planting dates and fertiliser application. The study realised limited research related to integrating seasonal forecasts into crop models for policy development. Widening of the interdisciplinary nature and scope of the studies through involvement of social scientists, as well as agricultural economists, will improve the scope and aim of such studies to policymaking. Application of research towards policymaking is critical for governments to steer human and financial resources towards application of integrated crop and seasonal forecast information. This can be beneficial in smallholder farming systems, which are the most vulnerable to climate fluctuations.

In comparison to statistical crop models, mechanistic crop models such as DSSAT are mostly used because they predict outputs of interest to both researchers and farmers within reasonable means and account for most farm management practices that can be used in climate variability management. Unsurprisingly, the use of GCM and statistical approaches such as analogue techniques are the most common in studies because they are more feasible and effective in linking seasonal forecasts and crop models compared to stochastic disaggregation.

Research on the integration of seasonal forecast and crop models potentially allows for the preliminary assessment of climate risks to farmers, prior to the beginning of the farming seasons and for equipping farmers with tools and knowledge to make key decisions for their farm management, given certain specific seasonal forecast information. Variation in seasonal forecast data as well as differences in specialities of crop models warrants the use of multiple seasonal forecast and crop models to enhance reliability of the outputs. Assessments can be undertaken under smallholder farming conditions who can potentially benefit more from enhanced decision-making to manage climate variability. Local agricultural extension workers can be the bridge between researchers and farmers to improve the understanding and dissemination of research outputs.

In conclusion, the most appropriate approach of integrating seasonal forecasts into crop models differs depending on the aim of the end user. If, for example, the aim is to yield prediction at the seasonal scale, all models are suitable, and statistical models would prove easier and often sufficiently competent under historical conditions. Conversely, if the aim is to assess different conditions such as different practices, varieties, or climate regimes, mechanistic crop models such as DSSAT are preferred. The crop model selection will also constrain the need for – and consequent choice of – seasonal forecasts’ temporal and spatial resolution, and often the seasonal forecast approach will be used. Accessibility and other technical characteristics (e.g., temporal and spatial horizons) will be considered in selecting a source of seasonal forecast. In all cases, seasonal forecast skill is known to vary with location.
